# Surgical resident experience with common bile duct exploration and assessment of performance and autonomy with formative feedback

**DOI:** 10.1186/s13017-023-00480-0

**Published:** 2023-02-06

**Authors:** Molly Q. Nyren, Amanda C. Filiberto, Patrick W. Underwood, Kenneth L. Abbott, Jeremy A. Balch, Francesca Dal Mas, Lorenzo Cobianchi, Philip A. Efron, Brian C. George, Benjamin Shickel, Gilbert R. Upchurch, George A. Sarosi, Tyler J. Loftus

**Affiliations:** 1 University of Florida College of Medicine, Gainesville, FL USA; 2 Department of Surgery, University of Florida Health, PO Box 100286, Gainesville, FL 32610 USA; 3 Department of Management, Ca’ Foscari University of Venice, Venice, Italy; 4 Department of Clinical, Diagnostic and Pediatric Sciences, University of Pavia, Pavia, Italy; 5 IRCCS Policlinico San Matteo Foundation, General Surgery, Pavia, Italy; 6 Department of Surgery, University of Michigan, Ann Arbor, MI USA; 7 Department of Biomedical Engineering, University of Florida, Gainesville, FL USA

**Keywords:** Common bile duct exploration, Surgery, Feedback, Performance, Autonomy

## Abstract

**Background:**

Common bile duct exploration (CBDE) is safe and effective for managing choledocholithiasis, but most US general surgeons have limited experience with CBDE and are uncomfortable performing this procedure in practice. Surgical trainee exposure to CBDE is limited, and their learning curve for achieving autonomous, practice-ready performance has not been previously described. This study tests the hypothesis that receipt of one or more prior CBDE operative performance assessments, combined with formative feedback, is associated with greater resident operative performance and autonomy.

**Methods:**

Resident and attending assessments of resident operative performance and autonomy were obtained for 189 laparoscopic or open CBDEs performed at 28 institutions. Performance and autonomy were graded along validated ordinal scales. Cases in which the resident had one or more prior CBDE case evaluations (*n* = 48) were compared with cases in which the resident had no prior evaluations (*n* = 141).

**Results:**

Compared with cases in which the resident had no prior CBDE case evaluations, cases with a prior evaluation had greater proportions of practice-ready or exceptional performance ratings according to both residents (27% vs. 11%, *p* = .009) and attendings (58% vs. 19%, *p* < .001) and had greater proportions of passive help or supervision only autonomy ratings according to both residents (17% vs. 4%, *p* = .009) and attendings (69% vs. 32%, *p* < .01).

**Conclusions:**

Residents with at least one prior CBDE evaluation and formative feedback demonstrated better operative performance and received greater autonomy than residents without prior evaluations, underscoring the propensity of feedback to help residents achieve autonomous, practice-ready performance for rare operations.

**Supplementary Information:**

The online version contains supplementary material available at 10.1186/s13017-023-00480-0.

## Background

Choledocholithiasis occurs in approximately 8–20% of patients with cholelithiasis and confers increased risk of potentially life-threatening cholangitis and biliary pancreatitis. Patients with choledocholithiasis typically undergo a two-stage approach of cholecystectomy and pre- or postoperative endoscopic retrograde cholangiography (ERC) [1]. Alternatively, a single- stage approach of a laparoscopic common bile duct exploration (CBDE) performed in conjunction with laparoscopic cholecystectomy is associated with greater technical success rates [2, 3], decreased hospital length of stay [2, 4], and fewer retained stones at follow-up evaluation [5], and similar rates of morbidity and mortality compared with the two-stage approach [6, 7].

Despite its value, many surgeons are uncomfortable performing CBDE, and its use is declining in the USA [8]. As a consequence, surgical resident exposure to CBDE is declining [9]. This trend is consistent with evidence that graduating residents are performing fewer cases than in previous eras and have lower confidence in their operative abilities [10–12]. Feedback, of which there are many forms, has been shown to improve procedure performance in multiple specialties and levels of training [13]. Given the reduction in CBDE exposure, it is increasingly important to benchmark the operative abilities of general surgery residents in performing CBDE and to maximize the odds that trainees will achieve autonomous, practice-ready performance.

Using validated operative performance and autonomy metrics reported by both residents and attending surgeons for 189 laparoscopic or open CBDEs performed at 28 institutions, this study tests the hypothesis that receipt of one or more prior CBDE operative performance assessments, accompanied by formative feedback, is associated with greater resident performance and autonomy.

## Methods

### Study design and data source

This observational study used an existing multicenter dataset maintained by the Society for Improving Medical Professional Learning (SIMPL) collaborative, which maintains a database of resident and attending surgeon workplace-based assessments of resident operative performance and autonomy, in association with case complexity and verbal feedback from attendings, for a wide range of surgical procedures (described in detail at: https://www.simpl.org/simpl, accessed 6 September 2022). Residents and attendings use a smartphone app to generate these assessments, and attending surgeons who complete the assessments have the option of dictating feedback. With increasing interest in competency-based performance assessment in surgical education, the advent of a smartphone workplace-based assessment application from SIMPL has offered opportunities to better understand the progression of resident operative performance and autonomy [14].

The SIMPL dataset for this study included trainee postgraduate year, procedure type, resident and attending ratings of operative performance and autonomy, resident and attending ratings of case complexity, and attendings’ dictated feedback and debriefing narratives. We included evaluations for CBDEs (*n* = 189) that did not involve hepaticojejunostomy or choledochoenterostomy and then divided assessments into two groups: cases in which the resident had no prior CBDE evaluations (*N* = 141) and cases in which the resident had one or more CBDE evaluations (*N* = 48). The University of Florida Institutional Review Board approved this study (IRB# 202,200,845). This study was performed in accordance with the Strengthening the Reporting of Observational Studies in Epidemiology (STROBE) reporting guidelines (see Additional File 1: Table S1).

### Operative performance, autonomy, and complexity assessments

SIMPL allows trainees and attending surgeons to assess trainee performance on five ordinal variables: critical deficiency, inexperienced with procedure, intermediate performance, practice-ready performance, and exceptional performance [14, 15]. Resident autonomy is quantified by the Zwisch Scale, consisting of four additional ordinal levels: show and tell, active help, passive help or supervision only [14, 16, 17]. The Zwisch Scale has been shown to be a valid and reliable way to differentiate faculty guidance levels provided from which to infer resident autonomy [14, 16, 17]. Operative cases were assessed by trainee and attending surgeon perception of case complexity relative to similar procedures: easiest third, average complexity, or hardest third [14]. Global scores for case complexity, performance, and autonomy were calculated as the mean between resident and attending assessments of each outcome with equal weight given to both evaluators.

### Sentiment analysis

This study builds on previous work with Natural Language Processing (NLP) and applies advanced sentiment analysis techniques using deep learning methods to assess the degree of positivity or negativity in dictated feedback. Operative performance assessments by attendings were analyzed by NLP techniques, which allowed for systematic evaluation of dictated feedback to understand overall sentiment. Sentiment analysis is a subtask of NLP encompassing the extraction of opinions, evaluations, attitudes, and emotions from written language [18]. This methodology offers the potential for greater contextual understanding of language compared with classical approaches to sentiment analysis which utilize linguistic analysis or rule-based phrasing match against predefined positive and negative work lists [19].

Machine learning models are pre-trained on large bodies of generalized human language (e.g., Wikipedia) and then fine-tuned on a task-specific dataset, which generate performance advantages for multiple NLP tasks [20–22]. We completed sentiment analysis of SIMPL dictations using Python version 3.8.8, the PyTorch machine learning framework, the Huggingface library, and an established deep learning model that was fine-tuned on the Stanford Sentiment Treebank v2 [23] dataset of movie reviews for predicting positive and negative sentiment from language [24, 25]. To address limitations in the pre-trained model, dictations were truncated to 128 tokens [26]. For each transcription, the deep learning model predicted a sentiment label (positive or negative) and a corresponding sentiment score ranging from 0.5 to 1.0. For dictations expected to be negative, sentiment scores were subtracted from 1.0 so that all transcription scores ranged from 0 (most negative) to 1 (most positive). Examples of positive and negative sentiment language in dictations can be found in Additional File 2: Table S2.

### Subgroup analysis of laparoscopic cases

The primary analysis of all CBDE cases demonstrated that there was a significantly greater proportion of laparoscopic cases performed by residents with prior CBDE evaluations. Therefore, we performed a subgroup analysis of laparoscopic CBDE cases, and repeated all elements of the primary analysis in this subgroup analysis of 46 cases of laparoscopic CBDE in which the resident had a prior evaluation versus the 100 cases of laparoscopic CBDE in which the resident had no prior evaluations. A visual flowchart of laparoscopic and open cases is illustrated in Additional File 3: Fig. S1.

### Statistical analyses

**Fig. 1:**
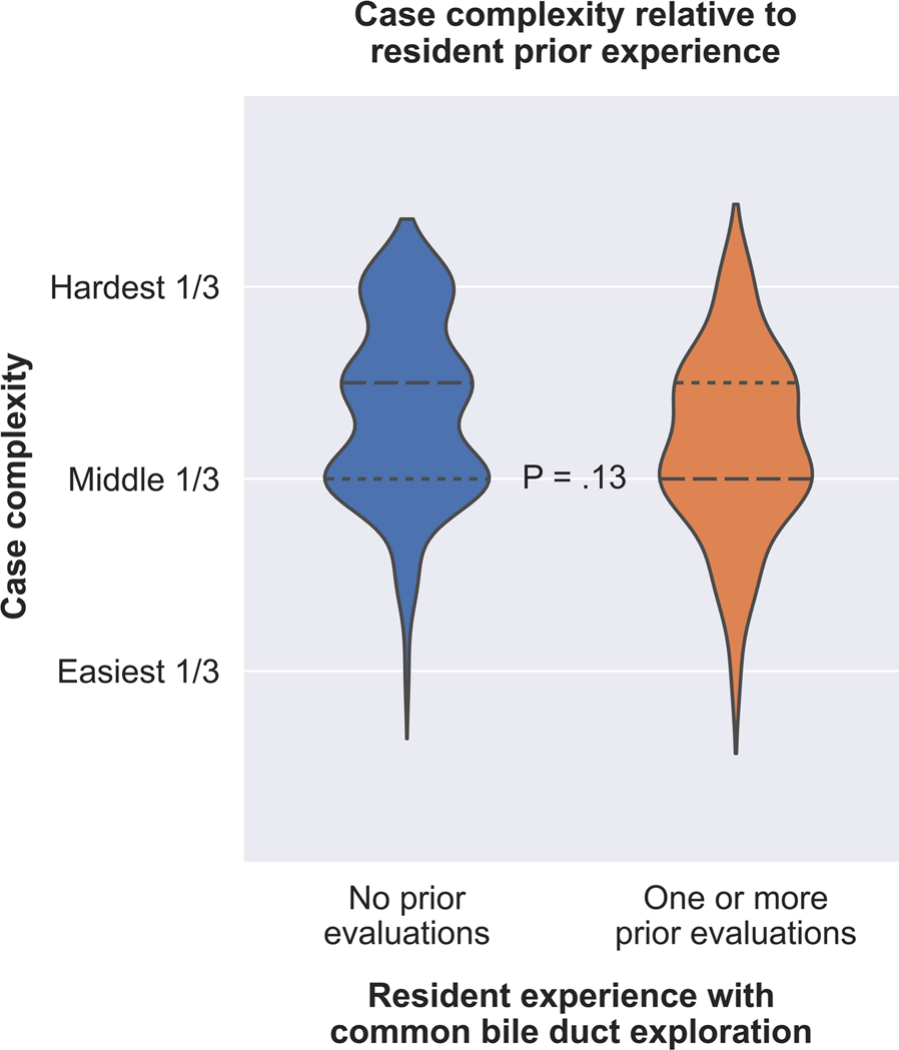
Case complexity for common bile duct explorations was similar between cohorts in which the resident had no prior cases with formative feedback versus one or more prior cases with formative feedback. Long dashes represent the median value. Short dashes represent the 25th and 75th percentiles

**Fig. 2:**
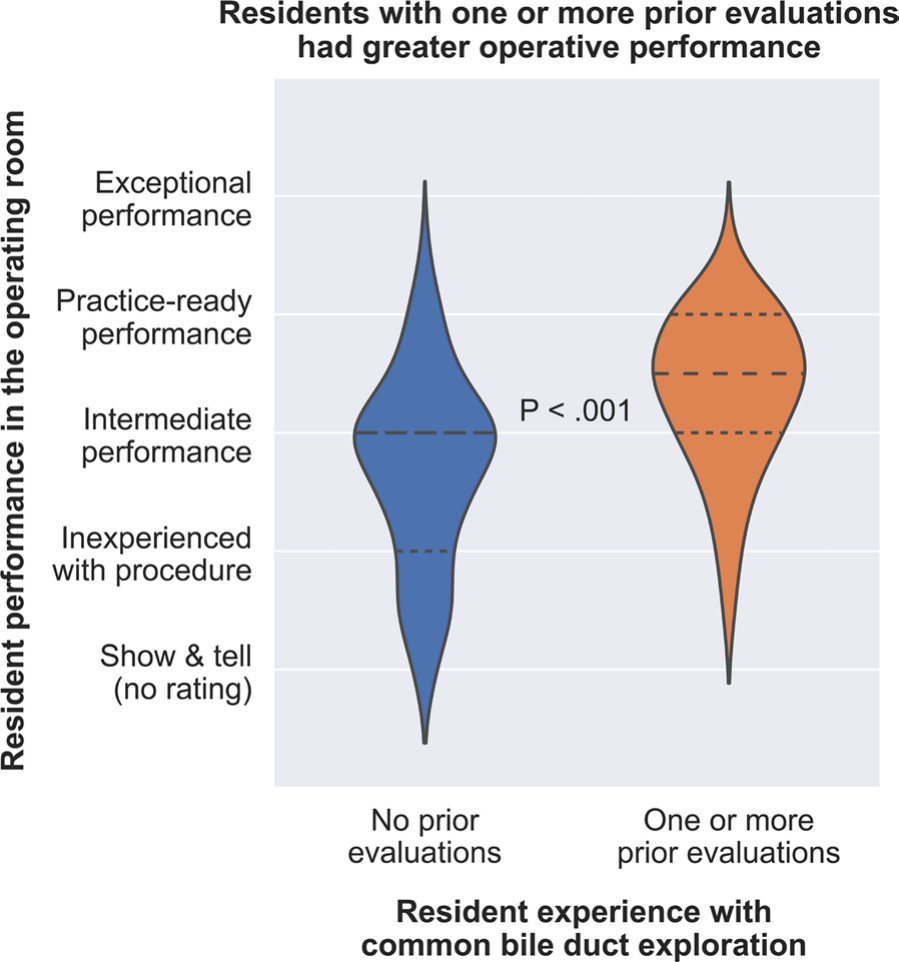
Operative performance was greater among residents who had performed one or more prior cases of common bile duct exploration with formative feedback compared with residents who had no prior cases with formative feedback. Long dashes represent the median value. Short dashes represent the 25th and 75th percentiles. For “Show and tell” cases in which the attending performs the case, there was no resident performance rating

**Fig. 3:**
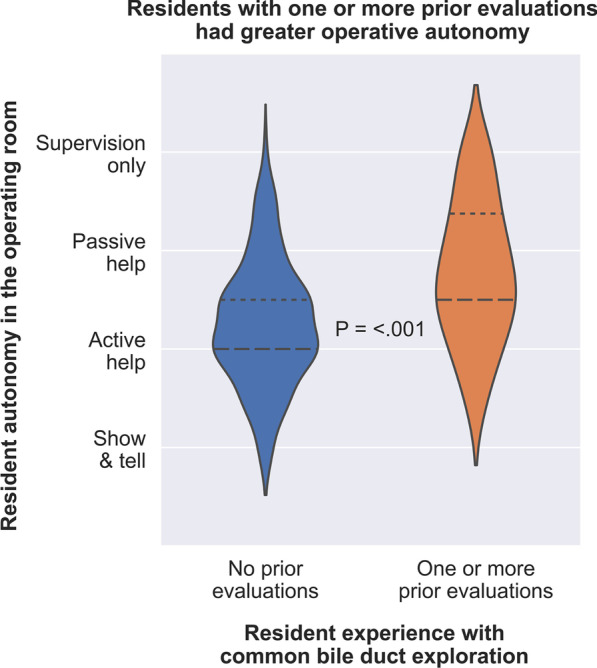
Operative autonomy was greater among residents who had performed one or more prior cases of common bile duct exploration with formative feedback compared with residents who had no prior cases with formative feedback. Long dashes represent the median value. Short dashes represent the 25th and 75th percentiles

Categorical variables were compared by Fisher’s exact test and presented as frequencies with percentages. Continuous variables were compared by the Kruskal–Wallis test and presented as median values with interquartile ranges (IQRs). All statistical tests were 2-sided with an alpha of 0.05. Given the lack of prior studies testing our hypothesis, all analyses were performed in an exploratory fashion. All primary analysis outcomes (i.e., those listed in Table 2 and the *p* values reported in Figs. 1, 2, and 3) were adjusted for multiple comparisons by performing Benjamini–Hochberg corrections.

## Results

### Case characteristics and complexity

We analyzed performance and autonomy ratings for 189 CBDEs performed by 92 residents who were evaluated by 64 attending surgeons at 28 institutions. Of 189 CBDEs, 141 (75%) had no prior CBDE performance assessments for that resident and 48 (25%) had at least one prior CBDE performance assessment, including 31 with one prior assessment, ten with two prior assessments, four with three prior assessments, and one with five prior assessments. In 12 of those 48 cases (25%), the resident was performing a CBDE with the same attending that had previously provided a CBDE evaluation for the resident. Of 189 CBDEs, 146 (77%) were laparoscopic and 43 (23%) involved an open approach. Residents with at least one prior CBDE evaluation had a greater proportion of laparoscopic CBDEs compared with the group without prior evaluations (96% vs. 71%, *p* < 0.001). Resident assessments of case complexity were similar between cases with and without prior evaluations; for attending assessments, there was a greater proportion of hardest third complexity cases in the no prior evaluations group (41.8% vs. 20.8%, *p* = 0.009). Averaging resident and attending assessments to calculate global assessments of case complexity demonstrated that the distribution of case complexity scores was higher in the no prior evaluations group, though the difference was not statistically significant (*p* = 0.13, Fig. 1, Table 1).

**Table 1:** Common bile duct exploration case characteristics stratified by resident experience

Case characteristics, *n* (%)	No prior evaluations (*n* = 141)	One or more prior evaluations (*n* = 48)	*p*
*Resident postgraduate year*			
1	10 (7.1)	0 (0.0)	.07
2	31 (22.0)	11 (22.9)	> .99
3	29 (20.6)	6 (12.5)	.28
4	25 (17.7)	13 (27.1)	.21
5	46 (32.6)	18 (37.5)	.60
*Resident gender*			
Female	53 (37.6)	15 (31.2)	.49
Male	84 (59.6)	33 (68.8)	.30
Unknown	4 (2.8)	0 (0.0)	.57
*Attending gender*			
Female	15 (10.6)	11 (22.9)	.05
Male	123 (87.2)	37 (77.1)	.11
Unknown	3 (2.1)	0 (0.0)	.57
*Operative approach*			
Laparoscopic	100 (70.9)	46 (95.8)	**< .001**
Open	41 (29.1)	2 (4.2)	**< .001**
*Resident assessment of case complexity*			
Easiest third	4 (2.8)	3 (6.2)	.37
Average complexity	74 (52.5)	25 (52.1)	> .99
Hardest third	37 (26.2)	14 (29.2)	.71
Missing	26 (18.4)	6 (12.5)	.38
*Attending assessment of case complexity*			
Easiest third	11 (7.8)	8 (16.7)	.10
Average complexity	71 (50.4)	30 (62.5)	.18
Hardest third	59 (41.8)	10 (20.8)	**.009**

Bold indicates statistically significant values

### Operative performance and autonomy

Resident and attending assessment of trainee performance and autonomy were compared between residents with one or more prior evaluations and residents without prior evaluations (Table 2). Attending and trainee assessments were averaged to calculate global assessments of operative performance and autonomy (Figs. 2 and 3). Residents with at least one prior CBDE evaluation had a greater proportion of practice-ready or exceptional performance ratings. Only 15 of 151 (11%) residents with no prior CBDE evaluations rated themselves as practice-ready or better, while 13 of 48 (27%) residents with at least one prior CBDE evaluation rated themselves as practice-ready or better (*p* = 0.02). By contrast, attendings rated 27 of 141 (19%) residents with no prior CBDE evaluations as practice-ready or better, and rated 28 of 48 (58%) residents with at least one prior CBDE performance assessment as practice-ready or better (*p* < 0.001). Globally, one or more prior evaluations were associated with significantly greater operative performance (*p* < 0.001), as illustrated in Fig. 2.

**Table 2:** Resident operative performance and autonomy during common bile duct exploration stratified by resident experience

Evaluation results, *n* (%)	No prior evaluations (*n* = 141)	One or more prior evaluations (*n* = 48)	*P*
*Resident assessment of resident performance*			
Critical deficiency	18 (12.8)	2 (4.2)	.21
Inexperienced with procedure	27 (19.1)	4 (8.3)	.21
Intermediate	55 (39.0)	23 (47.9)	.44
Practice-ready or exceptional	15 (10.6)	13 (27.1)	**.02**
Practice-ready	15 (10.6)	13 (27.1)	**.02**
Exceptional	0 (0.0)	0 (0.0)	> .99
Missing	26 (18.4)	6 (12.5)	.49
*Attending assessment of resident performance*			
Critical deficiency	20 (14.2)	3 (6.2)	.33
Inexperienced with procedure	25 (17.7)	5 (10.4)	.41
Intermediate	69 (48.9)	12 (25.0)	**.02**
Practice-ready or exceptional	27 (19.1)	28 (58.3)	**< .001**
Practice-ready	21 (14.9)	26 (54.2)	**< .001**
Exceptional	6 (4.3)	2 (4.2)	> .99
*Resident assessment of resident autonomy*			
Show and tell	18 (12.8)	2 (4.2)	.21
Active help	63 (44.7)	22 (45.8)	> .99
Passive help	28 (19.9)	10 (20.8)	> .99
Supervision only	6 (4.3)	8 (16.7)	**.02**
Missing	26 (18.4)	6 (12.5)	.49
*Attending assessment of resident autonomy*			
Show and tell	20 (14.2)	3 (6.2)	.33
Active help	76 (53.9)	12 (25.0)	**.004**
Passive help	34 (24.1)	22 (45.8)	**.02**
Supervision only	11 (7.8)	11 (22.9)	**.02**
*Attending verbal feedback sentiment*			
Verbal feedback was provided	66 (46.8)	21 (43.8)	.90
Sentiment score, median [interquartile range]	1.0 (0.0–1.0)	0.8 (0.0–1.0)	.44
Sentiment was positive	40 (60.6)	2 (57.1)	.94

Bold indicates statistically significant values

Residents with at least one prior CBDE evaluations were granted more operative autonomy than residents with no prior CBDE evaluations as assessed by both resident and attending (Table 2 and Fig. 3). Of 48 CBDEs performed by residents with at least one prior CBDE evaluation, 8 (17%) reported needing only supervision, rather than passive help, active help, or demonstration, compared to 6 of 141 (4.3%) CBDEs performed by residents with no prior CBDE evaluation (*p* = 0.02). Similarly, attendings reported only providing supervision for 11 of 48 (23%) residents with at least one prior CBDE evaluation, compared to 11 of 141 (8%) residents with no prior CBDE evaluation (*p* = 0.02). Globally, one or more prior evaluations were associated with significantly greater operative autonomy (*p* < 0.001), as illustrated in Fig. 3.

### Dictated feedback

Dictated feedback was present for 87 of 189 (46%) of CBDE evaluation, with no significant difference between assessments for residents with no prior CBDE evaluation and residents with at least one prior CBDE evaluation (Table 2). Sentiment scores for dictated feedback were also comparable for residents with (median 0.8, IQR 0.0–1.0) and without (median 1.0, IQR 0.0–1.0) prior CBDE evaluations (*p* = 0.44).

### Subgroup analysis of laparoscopic cases: case characteristics and complexity

LCBDE case characteristics, including resident postgraduate year, resident and attending gender, and case complexity, as evaluated by residents and attendings, were compared between residents with (*N* = 46) and without (*N* = 100) prior LCBDE evaluations (see Additional File 4: Table S3). Like results for all CBDEs, resident assessments of case complexity were similar between cases with and without prior evaluations; for attending assessments, there was a greater proportion of hardest third complexity cases in the no prior evaluations group (44.0% vs. 19.6%, *p* = 0.01). Averaging resident and attending assessments to calculate global assessments of case complexity demonstrated that the distribution of case complexity scores was higher in the no prior evaluations group (*p* = 0.05, see Additional File 5: Fig. S2).

### Subgroup analysis of laparoscopic cases: operative performance and autonomy

In the subgroup of LCBDE cases, among residents with prior evaluations, 29% rated themselves as practice-ready, while only 11% of residents without prior evaluations evaluated themselves as practice-ready (*p* = 0.007, see Additional File 6: Table S4). Similarly, attendings rated 59% of residents with prior evaluations as being practice-ready or exceptional, while rating only 18% of residents without prior evaluations as practice-ready or exceptional (*p* < 0.001). Globally, one or more prior evaluations were associated with significantly greater operative performance (*p* < 0.001), consistent with the primary analysis (see Additional File 7: Fig. S3).

Residents with prior LCBDE evaluations were granted more operative autonomy, as assessed by both residents and attendings. Seventeen percent of residents with prior evaluations rated themselves as receiving supervision only during LCBDE (the greatest amount of autonomy), while only 6% of residents without prior evaluations rated themselves as receiving supervision only (*p* = 0.04, see Additional File 6: Table S4). Attendings rated 70% of residents with prior evaluations as receiving passive help or supervision only, while 34% of residents without prior evaluations experienced similar levels of autonomy (*p* = 0.02). Globally, one or more prior evaluations in LCBDE were associated with significantly greater operative autonomy (*p* < 0.001, see Additional File 8: Table S4).

## Discussion

In this analysis of 189 CBDEs performed at 28 institutions, residents who had performed at least one prior CBDE with an evaluation demonstrated better operative performance and received greater autonomy than residents who had no prior CBDE evaluations. Nearly two-thirds of the residents in the prior evaluation group had only one prior evaluation, suggesting that the observed associations may be realized with minimal prior experience in the context of formative feedback; 75% of the residents in the prior evaluation group had never received formative feedback from the same attending, suggesting that gains in operative ability were transferrable between resident-attending teams. In this study, formative feedback was provided as numerical performance and autonomy assessments in all cases, as well as dictated feedback for almost half of all cases. Attendings (but not residents) rated CBDEs as more complex when the resident had no prior CBDE performance assessments, suggesting that attending surgeons perceived cases as more complex when the trainee is inexperienced. Sentiment analyses demonstrated that positivity in recorded, verbal feedback was similar between groups, suggesting that attending mood and positivity—which could be affected by resident experience level and performance—did not drive the observed associations between prior evaluations, performance, and autonomy. Finally, subgroup analyses demonstrated that prior CBDE experience with formative feedback was associated with greater performance and autonomy regardless of whether an open or laparoscopic approach was used.

Contributors to resident performance have long been a topic of interest in peer-reviewed literature. Prior studies have demonstrated an improvement in operative efficiency or error rate with formative feedback [13, 27–29]. Peer-reviewed literature on resident performance shows a positive association between postgraduate year and operative performance and autonomy [30]. In practice, surgeons with higher case volumes and experience are noted to have shorter duration of surgery and improved performance and patient outcomes [31, 32]. With respect to trainees, studies suggest that residents might be underprepared for independence with certain operations, especially when rarely performed [33, 34]. Despite the recent growth of more efficient forms of evaluation, such as via the SIMPL application, there have been no studies demonstrating improvement in operative performance and autonomy with operative evaluations and feedback.

There is limited trainee exposure to CBDE, with graduating general surgery residents performing one CBDE on average, and concern for declining operative autonomy for surgical residents [8–12, 35]. With these limitations, best practices for resident education and training become increasingly important. This study is unique as no study, to our knowledge, has shown that one or more prior receipt of a CBDE evaluation with formative feedback is associated with higher operative performance and autonomy. These data on CBDE learning curves provide an important quantitative benchmark against which other forms of experience, i.e., those not in the operating room, may be tested.

In recent years, the popularity of simulation courses to fill this educational gap has grown. Prior studies support the value of simulation, especially in the context of laparoscopic surgery [36–39]. There have been prior studies of the efficacy of simulation training in CBDE, yet it remains unclear whether CBDE skills gained in simulation transfer to the operating room [40–42]. Given the growing concern for resident case volumes and increased simulation opportunities, we suggest further evaluation of simulation in operative performance and autonomy, which may be assessed by SIMPL in a similar manner as done in this study.

### Limitations

This study is agnostic to resident prior experience with CBDE and formative feedback that occurred outside the SIMPL app. We acknowledge that residents with no prior evaluations may have undocumented experience, and residents with prior evaluations may have more experience than reflected in the SIMPL app. This undocumented exposure is a significant confounder for this study, but it is not captured by the SIMPL app at this time. We have little reason to suspect that unmeasured prior experience and formative feedback would be different between cohorts, and any such unmeasured events would likely serve to dilute the strength of associations in our study and generate false negative results, rather than generate false positive associations. Similarly, there is a possibly of attendings preferentially selecting residents to perform CBDE when they have performed this procedure before, suggesting that final results are skewed towards residents with more experience than those not represented in this study. However, we have little reason to believe this would change the outcome that evaluations are associated with increased performance and autonomy, if anything, a population of more experience residents would be less likely to show improvement. Another limitation is that our statistical analysis does not differentiate between trainees with greater than one prior evaluation and trainees with just one prior evaluation; doing so would decrease the size of the prior evaluation cohort by more than one third, and in the absence of prior work with which to perform a power analysis, we favored the larger sample size. With this concession the sample size remains low, but large enough to detect statistically significant differences in the primary outcome of resident operative performance and autonomy. Attending prior experience was not measured, and it is feasible that less experienced attendings were more likely to be operating with less experienced residents, which could affect the results. Similarly, there is a possibly of attendings preferentially selecting residents to perform CBDE when they have performed this procedure before, suggesting that results are skewed towards residents with more experience than expected. Finally, the observational design of this study does not establish causality, and we hesitate to use causal inference methods in the context of several unmeasured but potentially important variables.

## Conclusions

The single-stage approach to choledocholithiasis with CBDE is a clinically important procedure for which many surgical residents may not achieve practice-ready performance, due to limited case volumes. In this analysis of 189 CBDEs, residents who received one or more prior CBDE evaluations demonstrated better operative performance and experienced greater autonomy than residents with no prior CBDE evaluations. These findings underscore the value of feedback for helping surgical residents achieve autonomous, practice-ready performance for a rare operation.

## Supplementary Information

**Additional file 1**: **Table S1**. Table which illustrates the Strengthening the Reporting of Observational Studies in Epidemiology (STROBE) reporting guidelines

**Additional file 2**: **Table S2**. Table which illustrates examples of positive and negative language in dictations

**Additional file 3**: **Fig. S1**. Visual flowchart illustrating breakdown of cases, with 189 total cases performed with residents, 146 of which were laparoscopic and 43 were open. Of the 146 laparoscopic cases, 46 residents had one or more prior evaluations and 100 had no prior evaluations. Of the 43 open cases, 2 residents had one or more prior evaluations and 41 had no prior evaluations

**Additional file 4**: **Table S3**. Table illustrating subgroup analysis data for laparoscopic common bile duct exploration case characteristics stratified by resident experience

**Additional file 5**: **Fig. S2**. Figure illustrating that global case complexity for laparoscopic common bile duct explorations was higher in the cohort in which residents had no prior cases with formative feedback vs. one or more prior cases with formative feedback. Long dashes represent the median value. Short dashes represent the 25th and 75th percentiles.

**Additional file 6**: **Table S4**. Table illustrating subgroup analysis data for resident operative performance and autonomy during laparoscopic common bile duct exploration stratified by resident experience

**Additional file 7**: **Fig. S3**. Figure illustrating that global operative performance was greater among residents who had performed one or more prior cases of laparoscopic common bile duct exploration with formative feedback compared with residents who had no prior cases with formative feedback. Long dashes represent the median value. Short dashes represent the 25th and 75th percentiles.

**Additional file 8**: **Fig. S4**. Figure illustrating that global operative autonomy was greater among residents who had performed one or more prior cases of laparoscopic common bile duct exploration with formative feedback compared with residents who had no prior cases with formative feedback. Long dashes represent the median value. Short dashes represent the 25th and 75th percentiles.

## Data Availability

Data generated or analyzed during this study are included in this published article and its supplementary information files. Further information is available from the corresponding author on reasonable request.

## Abbreviations

CBDE: Common bile duct exploration
LCBDE: Laparoscopic common bile duct exploration
ERC: Endoscopic retrograde cholangiography
SIMPL: Society for Improving Medical Professional Learning
