# Supplementary material for: Surgical resident experience with common bile duct exploration and assessment of performance and autonomy with formative feedback
Source: World J Emerg Surg. 2023 Feb 6;18:13. doi: 10.1186/s13017-023-00480-0 (PMC9901129; doi:10.1186/s13017-023-00480-0)
Supplement: Supplementary file 1 — Additional file 1: Table S1. Table which illustrates the Strengthening the Reporting of Observational Studies in Epidemiology (STROBE) reporting guidelines [file 13017_2023_480_MOESM1_ESM.docx]

**Additional File 1. STROBE Statement Checklist for Cohort Study**

|  | | Item No | | Recommendation | Page No | |
| --- | --- | --- | --- | --- | --- | --- |
| **Title and abstract** | | 1 | | (*a*) Indicate the study’s design with a commonly used term in the title or the abstract | 2-3 | |
|  |  |  |  | (*b*) Provide in the abstract an informative and balanced summary of what was done and what was found |  | |
| Introduction | | | | | | |
| Background/rationale | | 2 | | Explain the scientific background and rationale for the investigation being reported | 4 | |
| Objectives | | 3 | | State specific objectives, including any prespecified hypotheses | 4-5 | |
| Methods | | | | | | |
| Study design | | 4 | | Present key elements of study design early in the paper | 5 | |
| Setting | | 5 | | Describe the setting, locations, and relevant dates, including periods of recruitment, exposure, follow-up, and data collection | 5-6 | |
| Participants | | 6 | | (*a*) Give the eligibility criteria, and the sources and methods of selection of participants. Describe methods of follow-up | 5 | |
|  |  |  |  | (*b*) For matched studies, give matching criteria and number of exposed and unexposed |  | |
| Variables | | 7 | | Clearly define all outcomes, exposures, predictors, potential confounders, and effect modifiers. Give diagnostic criteria, if applicable | 6 | |
| Data sources/ measurement | | 8* | | For each variable of interest, give sources of data and details of methods of assessment (measurement). Describe comparability of assessment methods if there is more than one group | 6-7 | |
| Bias | | 9 | | Describe any efforts to address potential sources of bias |  | |
| Study size | | 10 | | Explain how the study size was arrived at | 6-8 | |
| Quantitative variables | | 11 | | Explain how quantitative variables were handled in the analyses. If applicable, describe which groupings were chosen and why | 8 | |
| Statistical methods | | 12 | | (*a*) Describe all statistical methods, including those used to control for confounding | 7-8 | |
|  |  |  |  | (*b*) Describe any methods used to examine subgroups and interactions |  | |
|  |  |  |  | (*c*) Explain how missing data were addressed |  | |
|  |  |  |  | (*d*) If applicable, explain how loss to follow-up was addressed |  | |
|  |  |  |  | (*e*) Describe any sensitivity analyses |  | |
| Results | | | | |  | |
| Participants | | 13* | | (a) Report numbers of individuals at each stage of study—eg numbers potentially eligible, examined for eligibility, confirmed eligible, included in the study, completing follow-up, and analysed | 8 | |
|  |  |  |  | (b) Give reasons for non-participation at each stage |  | |
|  |  |  |  | (c) Consider use of a flow diagram |  | |
| Descriptive data | | 14* | | (a) Give characteristics of study participants (eg demographic, clinical, social) and information on exposures and potential confounders | 8 | |
|  |  |  |  | (b) Indicate number of participants with missing data for each variable of interest |  | |
|  |  |  |  | (c) Summarise follow-up time (eg, average and total amount) |  | |
| Outcome data | | 15* | | Report numbers of outcome events or summary measures over time | 8-11 | |
| Main results | 16 | | (*a*) Give unadjusted estimates and, if applicable, confounder-adjusted estimates and their precision (eg, 95% confidence interval). Make clear which confounders were adjusted for and why they were included | |  |  |
|  |  |  | (*b*) Report category boundaries when continuous variables were categorized | | 8-11 |  |
|  |  |  | (*c*) If relevant, consider translating estimates of relative risk into absolute risk for a meaningful time period | |  |  |
| Other analyses | 17 | | Report other analyses done—eg analyses of subgroups and interactions, and sensitivity analyses | | 11 |  |
| Discussion | | | | | |  |
| Key results | 18 | | Summarise key results with reference to study objectives | | 12 |  |
| Limitations | 19 | | Discuss limitations of the study, taking into account sources of potential bias or imprecision. Discuss both direction and magnitude of any potential bias | | 14 |  |
| Interpretation | 20 | | Give a cautious overall interpretation of results considering objectives, limitations, multiplicity of analyses, results from similar studies, and other relevant evidence | | 14-15 |  |
| Generalisability | 21 | | Discuss the generalisability (external validity) of the study results | | 13-14 |  |
| Other information | | | | | |  |
| Funding | 22 | | Give the source of funding and the role of the funders for the present study and, if applicable, for the original study on which the present article is based | | 16 |  |
