# Supplementary material for: Surgical resident experience with common bile duct exploration and assessment of performance and autonomy with formative feedback
Source: World J Emerg Surg. 2023 Feb 6;18:13. doi: 10.1186/s13017-023-00480-0 (PMC9901129; doi:10.1186/s13017-023-00480-0)
Supplement: Supplementary file 2 — Additional file 2: Table S2. Table which illustrates examples of positive and negative language in dictations [file 13017_2023_480_MOESM2_ESM.docx]

**Additional File 2. Sentiment in verbal dictations**

| **Example of negative dictation:** | **Example of positive dictation:** |
| --- | --- |
| “Regarding his laparoscopic ability [resident name] needs to improve upon using both hands to be efficient and smooth he seemed to be operating with only one hand his dominant hand while the other hand was not really helping.” | “Excellent job with this exploratory laparotomy and repair of duodenal perforation [resident name]'s tissue handling was excellent her overall performance in the operation showed that she understood the steps and she did not waste time she had good confidence and she was able to lead the intern through the critical steps of the operation.” |
