# Supplementary figures and images for: Surgical resident experience with common bile duct exploration and assessment of performance and autonomy with formative feedback
Source: World J Emerg Surg. 2023 Feb 6;18:13. doi: 10.1186/s13017-023-00480-0 (PMC9901129; doi:10.1186/s13017-023-00480-0)

## Slide 1
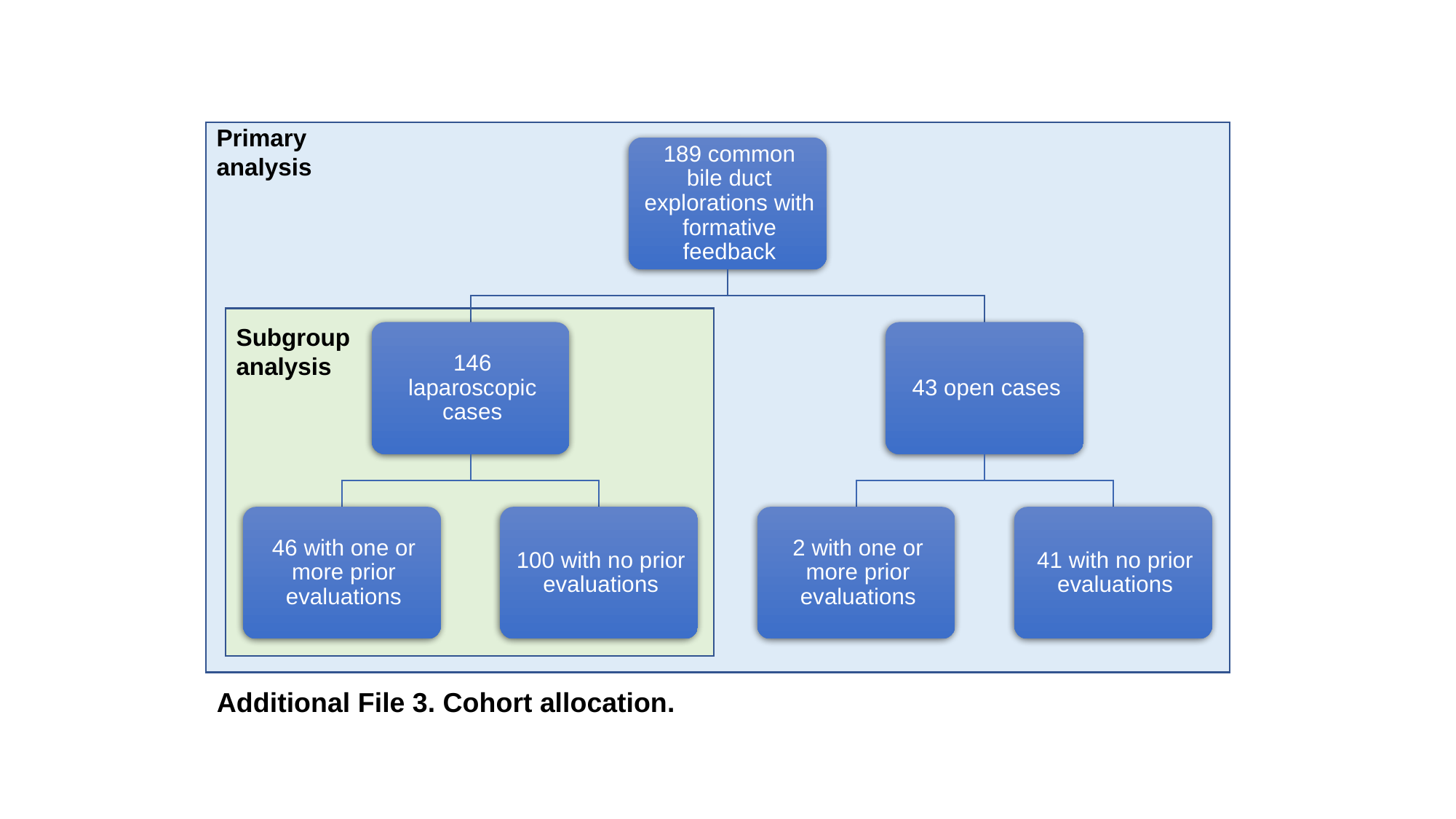

Primary
analysis
Subgroup
analysis
Additional File 3. Cohort allocation.

Supplement: Supplementary file 3 — Additional file 3: Fig. S1. Visual flowchart illustrating breakdown of cases, with 189 total cases performed with residents, 146 of which were laparoscopic and 43 were open. Of the 146 laparoscopic cases, 46 residents had one or more prior evaluations and 100 had no prior evaluations. Of the 43 open cases, 2 residents had one or more prior evaluations and 41 had no prior evaluations [file 13017_2023_480_MOESM3_ESM.pptx]

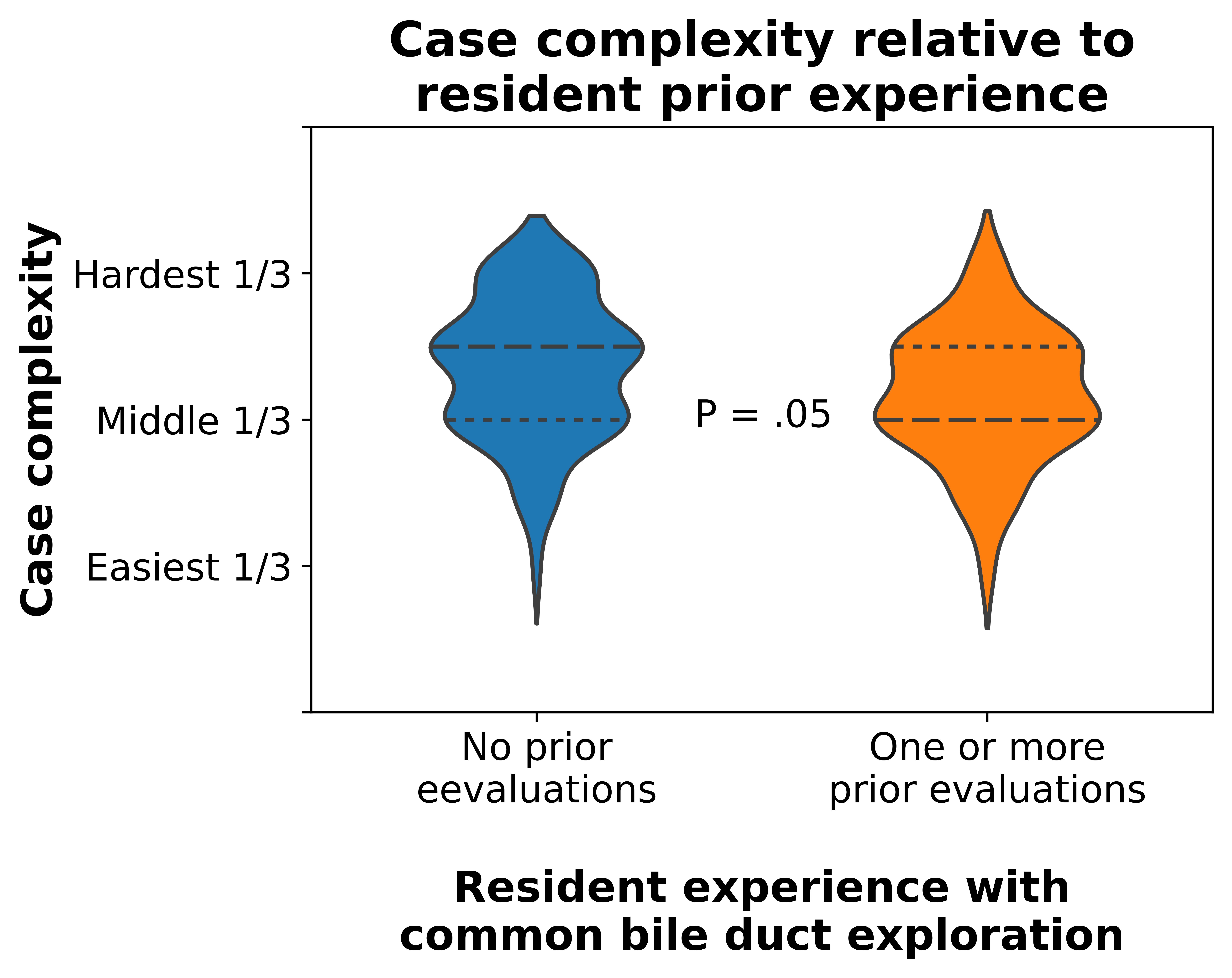

Supplement: Supplementary file 5 — Additional file 5: Fig. S2. Figure illustrating that global case complexity for laparoscopic common bile duct explorations was higher in the cohort in which residents had no prior cases with formative feedback vs. one or more prior cases with formative feedback. Long dashes represent the median value. Short dashes represent the 25th and 75th percentiles. [file 13017_2023_480_MOESM5_ESM.jpg]

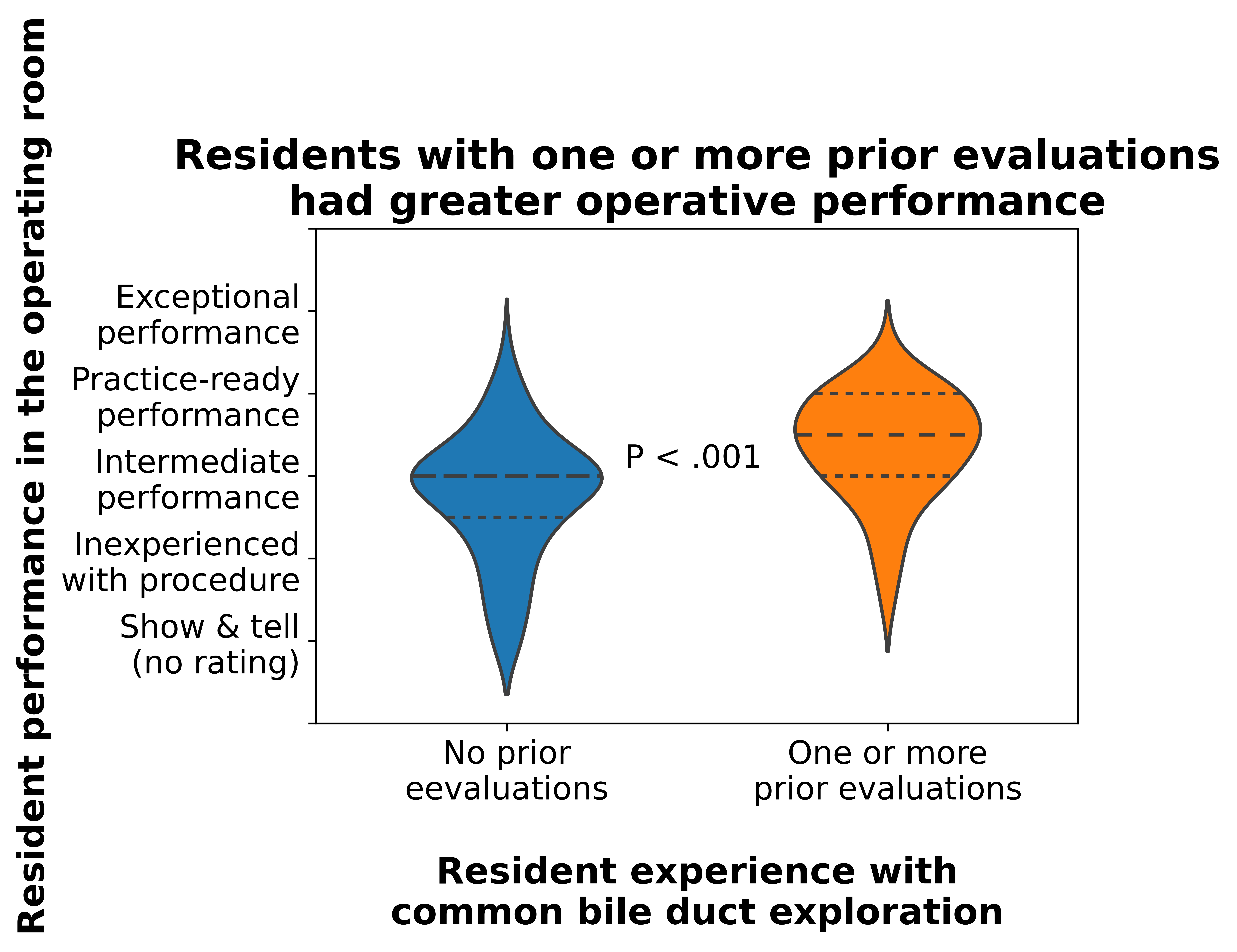

Supplement: Supplementary file 7 — Additional file 7: Fig. S3. Figure illustrating that global operative performance was greater among residents who had performed one or more prior cases of laparoscopic common bile duct exploration with formative feedback compared with residents who had no prior cases with formative feedback. Long dashes represent the median value. Short dashes represent the 25th and 75th percentiles. [file 13017_2023_480_MOESM7_ESM.jpg]

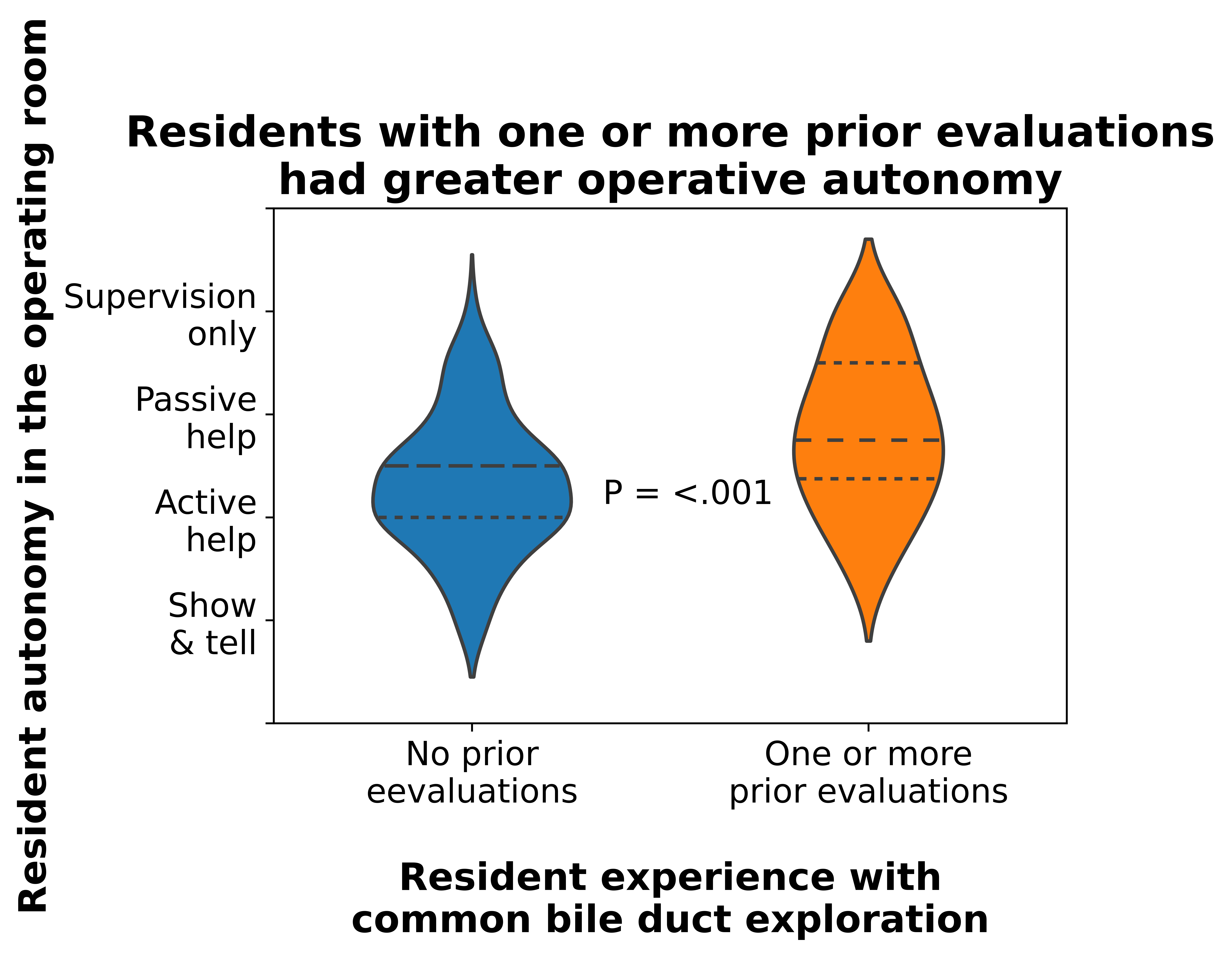

Supplement: Supplementary file 8 — Additional file 8: Fig. S4. Figure illustrating that global operative autonomy was greater among residents who had performed one or more prior cases of laparoscopic common bile duct exploration with formative feedback compared with residents who had no prior cases with formative feedback. Long dashes represent the median value. Short dashes represent the 25th and 75th percentiles. [file 13017_2023_480_MOESM8_ESM.jpg]
