# Supplementary material for: Surgical resident experience with common bile duct exploration and assessment of performance and autonomy with formative feedback
Source: World J Emerg Surg. 2023 Feb 6;18:13. doi: 10.1186/s13017-023-00480-0 (PMC9901129; doi:10.1186/s13017-023-00480-0)
Supplement: Supplementary file 4 — Additional file 4: Table S3. Table illustrating subgroup analysis data for laparoscopic common bile duct exploration case characteristics stratified by resident experience [file 13017_2023_480_MOESM4_ESM.docx]

**Additional File 4.** Laparoscopic common bile duct exploration case characteristics stratified by resident experience.

| **Case characteristics**, n (%) | **No prior evaluations**  (n=100) | **One or more prior evaluations**  (n=46) | **P** |
| --- | --- | --- | --- |
| **Resident postgraduate year** |  |  |  |
| 1 | 8 (8.0) | 0 (0.0) | .06 |
| 2 | 22 (22.0) | 11 (23.9) | .83 |
| 3 | 26 (26.0) | 6 (13.0) | .09 |
| 4 | 19 (19.0) | 13 (28.3) | .28 |
| 5 | 25 (25.0) | 16 (34.8) | .24 |
| **Resident gender** |  |  |  |
| Female | 43 (43.0) | 15 (32.6) | .28 |
| Male | 56 (56.0) | 31 (67.4) | .21 |
| Unknown | 1 (1.0) | 0 (0.0) | >.99 |
| **Attending gender** |  |  |  |
| Female | 15 (15.0) | 11 (23.9) | .24 |
| Male | 85 (85.0) | 35 (76.1) | .24 |
| Unknown | 0 (0.0) | 0 (0.0) | >.99 |
| **Resident assessment of case complexity** |  |  |  |
| Easiest third | 4 (4.0) | 3 (6.5) | .68 |
| Average complexity | 48 (48.0) | 24 (52.2) | .72 |
| Hardest third | 25 (25.0) | 13 (28.3) | .69 |
| Missing | 23 (23.0) | 6 (13.0) | .19 |
| **Attending assessment of case complexity** |  |  |  |
| Easiest third | 11 (11.0) | 8 (17.4) | .30 |
| Average complexity | 45 (45.0) | 29 (63.0) | **.05** |
| Hardest third | 44 (44.0) | 9 (19.6) | **.01** |
