# Supplementary material for: Surgical resident experience with common bile duct exploration and assessment of performance and autonomy with formative feedback
Source: World J Emerg Surg. 2023 Feb 6;18:13. doi: 10.1186/s13017-023-00480-0 (PMC9901129; doi:10.1186/s13017-023-00480-0)
Supplement: Supplementary file 6 — Additional file 6: Table S4. Table illustrating subgroup analysis data for resident operative performance and autonomy during laparoscopic common bile duct exploration stratified by resident experience [file 13017_2023_480_MOESM6_ESM.docx]

**Additional File 6.** Resident operative performance and autonomy during laparoscopic common bile duct exploration stratified by resident experience.

| **Evaluation results**, n (%) | **No prior evaluations**  (n=100) | **One or more prior evaluations**  (n=46) | **P** |
| --- | --- | --- | --- |
| **Resident assessment of resident performance** |  |  |  |
| Critical deficiency | 10 (10.0) | 2 (4.3) | .34 |
| Inexperienced with procedure | 16 (16.0) | 4 (8.7) | .31 |
| Intermediate | 41 (41.0) | 21 (45.7) | .72 |
| Practice-ready or exceptional | 10 (10.0) | 13 (28.3) | **.007** |
| Practice-ready | 10 (10.0) | 13 (28.3) | **.007** |
| Exceptional | 0 (0.0) | 0 (0.0) | >.99 |
| Missing | 23 (23.0) | 6 (13.0) | .19 |
| **Attending assessment of resident performance** |  |  |  |
| Critical deficiency | 15 (15.0) | 3 (6.5) | .18 |
| Inexperienced with procedure | 11 (11.0) | 4 (8.7) | .78 |
| Intermediate | 56 (56.0) | 12 (26.1) | **.001** |
| Practice-ready or exceptional | 18 (18.0) | 27 (58.7) | **<.001** |
| Practice-ready | 15 (15.0) | 25 (54.3) | **<.001** |
| Exceptional | 3 (3.0) | 2 (4.3) | .65 |
| **Resident assessment of resident autonomy** |  |  |  |
| Show & tell | 10 (10.0) | 2 (4.3) | .34 |
| Active help | 42 (42.0) | 21 (45.7) | .72 |
| Passive help | 19 (19.0) | 9 (19.6) | >.99 |
| Supervision only | 6 (6.0) | 8 (17.4) | **.04** |
| Missing | 23 (23.0) | 6 (13.0) | .19 |
| **Attending assessment of resident autonomy** |  |  |  |
| Show & tell | 15 (15.0) | 3 (6.5) | .18 |
| Active help | 51 (51.0) | 11 (23.9) | **.002** |
| Passive help | 26 (26.0) | 21 (45.7) | **.02** |
| Supervision only | 8 (8.0) | 11 (23.9) | **.02** |
| **Attending verbal feedback sentiment** |  |  |  |
| Verbal feedback was provided | 49 (49.0) | 20 (43.5) | .59 |
| Sentiment score, median [interquartile range] | 1.0 [0.0-1.0] | 0.7 [0.0-1.0] | .36 |
| Sentiment was positive | 31 (31.0) | 11 (23.9) | .59 |
